# The Urine and Its Derangements

**Published:** 1872-07

**Authors:** 


*The Urine and its Derangements. With the Application of Physiological Chemistry. To the Diagnosis and Treatment of the Constitutional as well as Local Diseases.* By GEORGE HARLEY, M. D., F. R. S. With Illustrations. Philadelphia: Lindsay & Blakiston, 1872.

Entirely agreeing with the Author that the Urine offers no royal road to knowledge of disease, we nevertheless think that a properly made examination of the urine offers a large and valuable field to the practitioner both in confirming and assisting in the diagnosis of disease.

Many of the so called handbooks have been so loaded with minute discussions in chemistry and urology as to make them altogether distateful and valueless to the general practitioner. From this cause the subject has not received that attention which practitioners otherwise would have desired to give it. The work of Dr. Harley takes up the subject in the easy and familiar style of the lecture room, which at once arrests the attention of the reader, making it both interesting and profitable.

The subject of urinary examinations is one that not only requires a preparation, but also careful and diligent application. To such as are willing to enter the investigation determined to make it thorough, the work of Dr. Harley will prove a valuable assistant and is truly an acquisition of great worth.

